# The Application of Disinfectants and Escharotics in the Treatment of Alveolar Abscess

**Published:** 1883-06

**Authors:** Frank Brewer


AMERICAN JOURNAL

OF

DENTAL SCIENCE.

VOL. XVII. THIRD SERIES—JUNE, 1883. No. 2.

ARTICLE I.

### IN THE TREATMENT OF ALVEOLAR ABSCESS, IS THE PASSAGE OF AN ESCHAROTIC, OR EVEN DISINFECTANT BEYOND THE TOOTH APICES IMPERATIVELY REQUISITE?

BY FRANK BREWER, SAN FRANCISCO, CAL.

We reply most negatively! and why? Not simply because we regard contact of all *undiluted* escharotics with the perice-mental (*i. e.*, periosteal) tissues most fatal to their integrity; but an experience in treating alveolar abscess, embracing a period of over a quarter of a century, particularly the more intimate during the last fifteen years, fully substantiates, in our mind, the fact that *all* cases curable through any form of medicinal application be it "escharotic," "disinfectant," or but a simple *deodorizer*, may become as fully susceptible through the mere influence of their *volatile* virtue as possible by contact of ingredient, *i. e.*, the usual form. Not only that, but as well have we found also, that the results, based by analogous comparisons, are more favorable to a permanent convalescence, and cases seemingly complicated become reduced to simplification.

So sensitive were we to the *permanent*, as well as temporary injuries evinced through contact of "carbolic acid"

or " creosote " with exterior human tissues, especially the more discernable with cases attempting their own palliation while laboring with " Odontalgia," that we determined over fifteen years since, when employing those ingredients in treating tooth-abscess, not only to dilute their power of causticity, but also modify the process of their application, by *invariably restricting* their presence *within the root canal* of the affected tooth, thus thereby entirely avoiding the most serious customary manual exercise of violently forcing, *i. e.*, pumping, the caustic in and upon most *vital* tissue! Hence we deem it proper to place upon record the fact, that during the period mentioned, not one drop of those fluids named, nor any other " caustic," " antiseptic," " disinfectant," or even " *deodorizer*," has been *intentionally* passed through, or beyond the apices of any tooth so affected; nor have we been *compelled* to so pass, even though our case presented advanced chronicity. But under all stages presenting a possibility of convalescence, and where the osseous walls remote from the alveolar parieties were not involved, the boundary line of lesionary termination has been reached! and we most unhesitatingly pronounce the mastery no more insurmountable than that with the usual course. This will also embrace conditions more advanced and deemed *permanently* incurable. But these were merely experimented upon in order to ascertain the extent of volatile efficacy upon conditions complicated. Cases successful, and we mean permanently so, or at least of six and seven years' standing, and now under our constant surveillance, will not only embrace those conditions termed blind abscesses, or those (in error) pronounced *self*-curable, but as well those presenting an abscence of a fractionary segment of cemental apicial periphery, as well as those whose chronicity extended through a period of from three to five years; and that, too, in several instances, where the exterior outlet of " fistulæ " was quite remote. The method of application of medicants as pursured by us consisted merely of a pledget of cotton placed at the apical third of the affected root, the canal being

*enlarged* when found *inadequate* to such admission, the dressings being changed as occasion required.' In the selection of a medicant, those customary escharotics, carbolic acid, or creosote, or again, that of iodine, were not alone indulged, but many far milder "disinfectants," and as well, "*dedorizers*" were favored, among which I mention chloride of lime water, Florida do., orange do., essence, as well as oil of lemon, same of cinnamon, sassafras, peppermint, essence of coffee (the latter equally efficient); again, paregoric, also "Eucalyptus," and what may perhaps prove more novel to my readers, simple dressings of unmedicated cotton! Though in its literal sense I stand corrected; for the cotton being absolutely absorbent, did contain an especial factor or, if you please, *two,* which in my most humble judgment, proved most efficient in neutralizing and diffusing the gasses constantly exuviating, following "stasis" and cystic degeneration, and which even though maxillary aperture be affected, and the apical root entrance be immediately sealed, *still remain exerting their toxic influence upon* the *involved tissues.* The factors alluded to (combined) were oxygen and hydrogen, or *atmosphere!* Although my reader may fail to perceive any especial healing virtue among many of the above ingredients named, still, nevertheless, the motive sought was fully achieved, and the hypothesis of the author as well confirmed to the effect that following elimination of the irritant, and diminishment of effusion and modification of toxic power incident to gasses, nature merely demands a free and uninterrupted outlet from the *focal* point of the *seat* of the disease to the exterior, not only during *stasis,* but throughout the entire process of cystic degeneracy, if such exist, or during the continuation of any discharge of *putrescent* purulent matter, or even inodorous fluids. And as the apex of a tooth leads more directly to that point, and as a natural aperture already exists and continues throughout the entire peduncular region, leading from the apices to the heart of the cysts, when such exist. And further, as Nature most invariably selects the canal of the root through which to dump her excretions, gasses included, the diameter of the apicial entrance permitting, even though an artificial maxillary sinus exist, most assuredly the channel would seem the most efficient through which to administer to the disease. The *capsular* type of pericemental formation and its immediate embracement of the fang area only adds to that hypothesis. We have invariably held to this method, and any case of failure to subdue the lesion has proved, by the alternate mode of administration, *i. e.*, by way of maxilla, fully as unsuccessful. My reader so far perceives that our dependence to success bases itself upon the *volatility* of the agent employed; but this is not all, nor the fundamental point of our argument. What we hold most prominently in view is the fact that *nature cures*, not man; and her reparative endowments and ready generosity are ever respondent, and fully effective, if all conditions adequate to other inflammatory conditions be but fully observed. To set aside an idea that through percolation, aided by gravitation and other causes, the medicant reached and acted upon the diseased tissues, (and this is possible with the inferior teeth,) we mention the fact that a majority of the cases operated upon embraced those of the *superior* arch; and as that old, yet trite " aphorism " will have it that " Water runs not up hill except per force ;" and again in most cases, as the dressings were merely volatilized, how, I pray, is it possible? Again, that the same did not embrace that class styled *self*-curable, conditions already enumerated fully offset, and we add thereto the fact that in our regular practice no root or tooth of possible utility has been taken from either arch, the tractionary force of which was inadmissible to finger and thumb, during the period mentioned!

In the one mouth so operated upon and now under our eye, thirteen cicatrices are traceable, eleven in the superior arch; in another, eleven; another, eight, another, five; and numerous cases of three and four in one arch, not to mention an abundance of cases of one and two. And as a period extending from two to seven years has already passed, without reverse manifestations, and that, too, in a climate favoring active progression from lesionary incipiency, we feel amply authenticated in a public announcement. That we might the better familarize our observation of a possible variation of ossific activity in the labor of sealing the apicial aperaturc by nature, several root canals were temporarily filled with gutta-percha, and the same examined at the expiration of three, six, twelve, and again at eighteen months. Those cases of spontaneous nerve devitalization, activity of ossific energy, proved the more prominent, and entire erasement in three cases occurred in three months, in two cases in which the remnant of a nerve was removed by barb (unarsenized) and when the point of the broach freely penetrated the apical aperature, the same became entirely obliterated in four months in one, and five months in the other. Those experimental upon and arsenic placed and sealed, not only in the month of the canals but at the apicial third, *failed* entirely, although unusual care was observed in the subsequent treatment to induce encouragement to erasure. Those cases in which the method was pursued in devitalizing the nerve, as invariably followed by the author, (and mentioned in a paper upon "Experimentation with arsenic upon the human tooth," at the last session of the California State Dental Association,) the activity of ossific deposition proved as favorable as those unarsenized. This method consists of merely puncturing the periphary with a sharp broach point previously dipped in cobalt and creosote or carbolic acid, to expedite, and where time may be limited, when in arsenic—but only when unavoidable. Sloughing *invariably* follows in from five to ten days. If found previously inflamed, treatment with anodyne and escharotic is observed, and painlessness of puncture encourged by an anæsthetic.

True, frequently is it found the fact that cases require quite protracted treatment before the lesion is subdued. This, in our mind arises through a similarity of idiosyncracies co-incident with other body abscesses, and the instance in many conditions is seized upon in reducing blood dyscrasia, and continues until parts remote are more or less relieved. In such instances dressings require frequent renewal, but medicinal administrations are entirely unnecessary, aside from that disinfectant and during the greater continuation of administration, oxygen is wholly sufficient, *i. e.*, simple, unmedicated absorbent cotton. Under conditions just named, *over treatment*, *i. e.*, feeding medicines in abundance (and I refer to the customary course of administration) is frequently the forerunner to *spoliation!* Experiments have plainly exhibited to us this fact, the fistulæ closing only upon cessation of interference, I know of no specialty as justifying in our profession as "Alveolar Abscess." To be successful one must possess or acquire an ardent love for his task, *arduous* though it be. He who marks time by the hour, or hastes to be rich, is no *true custodian* in this disease. Allow us to remark that although we failed in "cutting our eye teeth" until our tutelage under the original, and yet customary method of treating this lesion embraced a period of ten or twelve years, still we feel quite gratified by having grappled with *both sides* of the issue, and have well learned of the varied *eccentricities* habitual in "pericemental" lesions; also of the slight etiological sequences only necessary to derangement under loss of the more central support of the tooth organ; and the more *vividly* of the simple "*therapeutical*" aid only *demanded* by *nature* in recuperation. Of one fact we are fully assured, and that is that for the past twenty-five or thirty years, and meagre success of alveolar abscess in the hands of the best experts, may be distinctly traceable to an empirical indulgence of "*arsenic*." My experiments with this article, and the microscopic analysis of a human tooth by Messrs. Bodecker, Heitzman and Abbott, fully confirm it. Arsenic not only travels throughout the body of the nerve organ proper, but far beyond, destroying the minor nerve off-shoots of the maxillaries, which assist in nourishing the root investures,

(and which may be observed by careful dissection); but it does not stop at that, but also consumes all tubular investments; and what is still more fatal, (and the *sine qua non* to liquefaction of the sheath of the root,) it swallows up the "*cemental* vitalized *constituency*, thus instituting ossific liquefaction and osteomalacia. In summoning up our success in the treating of alveolar abscess, allow us to contribute the following reasons: 1st. The immediate correction of constipation, preference favoring saline correction rather than vegetable. 2nd. Sufficiently *enlarging* root canals to their *apices* whereby to readily and freely admit dressing, and the permanent sealing of the apicial aperature without disturbance of investure contiguous to and covering foramen. 3d. *Restriction* of all *medicants* within the root canal, and under no conditions disturbing the parts beyond. 4th. Change of dressings until a condition is *reached* defining the course terminating the disease, *i. e.*, *complete cessation* of all *fluid emanations*, putrescent, purulent, and *non-putrescent;* also, rest of tooth and freedom from undue concussion throughout recuperation. 5th. *Temporary fillings* for a period justifying full or at least greatly advanced restitution to a *possible normality.*

It requires but a mere cursory glance throughout our Dental literature of the present day to convince of the evident fact that but slight, if any modification, supplants the original method of treating this disease. From the earliest period of the area recognizing a possible success, when preceded by a certain course of treatment, the passage of creosote, carbolic acid, chloride of zinc, and as well even sulphuric acid, have been earnestly advocated, and most lavishly indulged, and that, too, regardless of the fact that, through loss of the *interior* support, the tooth organ is already most *prominently weakened*, and as well that the caustic properties must further deteriorate the anatomical relationship of the remaining tissue, and the vesicular accuracy of which is the more essential to its future nourishment. No sane man will attempt denial of the destructiveness of all four of the ingredients just mentioned when in contact with exterior human tissue. If such be due to atmospherical influence, why should not the same conditions arise at the apices? for upon nerve exposure is not the atmosphere admitted to that section, or the more readily and in abundance following absence of that organ? However, too much valuable space is already solicited from our *good editor*; hence, in conclusion, we present the following points for our future reflection:

1st. That through the presence of "cauterants" within the alveolar chamber, not alone are the fungoid anatomy and its nourishing plasma arrested, but likewise that part of the root investments which may be but incipiently affected, the same retaining an increased volume of the escharotic through its detachment from the fang area, and by which not alone the dermal, *i. e.*, ossific function is deteriorated, but also that of the subdermal formation, thus encouraging cicatricial tissue and evantuating in death, and liquefaction and osteomalacia of the cemental process throughout, at least at the apicial area.

2d. That cases are extremely *rare demanding* their presence in any form beyond the apices, fistulæ or otherwise, and they are permissable only where necrosis is positively affirmed.

3d. Owing to obscurity of the parts involved, no defined limits of diseased boundary is possible, nor can the maximum or minimum limits of "cystic" formation be accurately determined.—*Ohio State Journal of Dental Science.*

---

ARTICLE II.

